# Isolation and characterization of a novel *Tenacibaculum* species and a corresponding bacteriophage from a Mediterranean fish hatchery: Description of *Tenacibaculum larymnensis* sp. nov. and Tenacibaculum phage Larrie

**DOI:** 10.3389/fmicb.2023.1078669

**Published:** 2023-02-28

**Authors:** Maria Ioanna Tsertou, Adriana Triga, Stavros Droubogiannis, Constantina Kokkari, Grammatiki Anasi, Pantelis Katharios

**Affiliations:** ^1^Institute of Marine Biology, Biotechnology and Aquaculture, Hellenic Centre for Marine Research, Heraklion, Greece; ^2^Department of Biology, School of Sciences and Engineering, University of Crete, Heraklion, Greece; ^3^PHILOSOFISH S.A., Larymna, Greece

**Keywords:** phage-host system, *Tenacibaculum*, fish hatchery, bacteriophage, *Flavobacteriacae*

## Abstract

*Tenacibaculum larymnensis* sp. nov., a novel species of the *Tenacibaculum* genus was isolated from a commercial fish hatchery in Greece. The novel species is phylogenetically close to *T. discolor* and was biochemically and genetically characterized. The genome of *T*. *larymnensis* has 3.66 Mbps length, 31.83% GC content and the genomic analysis demonstrated that it harbors a wide enzymatic repertoire suggestive of increased degrading capacity but also several virulence factors including hemolysins, secretion systems, transporters, siderophores, pili and extracellular proteins. Using the novel strain, a virulent bacteriophage designated as Tenacibaculum phage Larrie was isolated and characterized. Larrie is a novel Siphovirus with relatively large genome, 77.5 kbps with 111 ORFs, a GC content of 33.7% and an exclusively lytic lifestyle. The new phage-host system can serve as an efficient model to study microbial interactions in the aquatic environment which contribute to the nutrient cycling.

## 1. Introduction

The genus *Tenacibaculum* contains 32 different species, 8 of which have been associated with disease in fish (Nowlan et al., 2020). Tenacibaculosis, the disease attributed to members of this genus affects a wide range of fish species including Atlantic salmon (*Salmo salar*) (Avendaño-Herrera et al., 2020; Lagadec et al., 2021), European seabass (*Dicentrarchus labrax*) (Avendaño-Herrera et al., 2006; Piñeiro-Vidal et al., 2012), sole (*Solea senegalensis*) (Avendaño-Herrera et al., 2006; Piñeiro-Vidal et al., 2012), and turbot (*Schophthalmus maximus*) (Piñeiro-Vidal et al., 2008c). Tenacibaculosis is mainly characterized by epidermal lesions and erosion of the skin and fins, while bacteria may colonize fish gills leading to breathing distress (Avendaño-Herrera et al., 2006; Frisch et al., 2018). Tenacibaculosis is one of the most serious diseases in the European seabass aquaculture and it is a growing concern also for the salmon aquaculture industry. *Tenacibaculum* spp. are common inhabitants of fish hatcheries (Timur and Yardımcı, 2015; Småge et al., 2016; Almeida et al., 2021). Even though the presence of certain species of known pathogenicity is alarming for most hatcheries, the actual role of these bacteria is yet far from being fully elucidated. They can be found in biofilters or in tank biofilms or even in the tank water.

Many members of the *Flavobacteriacae* display increased enzymatic activity and can degrade various polymers, polysaccharides, chitin, agar and cellulose and therefore contribute significantly to carbon cycling in the marine environment (Suzuki et al., 2001; Romero et al., 2014; Taylor et al., 2014; Almeida et al., 2021). Environmental *Tenacibaculum* spp. are enriched when there is polysaccharide availability either by taking advantage of other microorganisms’ enzymatic activity or due to their diverse and extensive carbohydrate-active enzymes (CAZymes) repertoire. They showcase a CAZyme-related niche specialization (Bunse et al., 2021). Pathogenic *Tenacibaculum* strains are usually found to be unable to utilize marine carbohydrates, even if they harbor CAZymes (Pérez-Pascual et al., 2017). Some pathogenic species are also lacking these genes completely, suggesting a diversion in niche exploitation (Bridel et al., 2018).

The importance of the microbial community in fish hatcheries to the health and welfare of fish has been recognized for many years now and several studies have been conducted to map these communities during healthy rearing conditions but also during disease outbreaks (Roalkvam et al., 2019; Levican et al., 2020). However, most if not all these studies focus exclusively on the bacterial part of the microbial community. Bacteriophages, the viruses of bacteria, are among the most important drivers of bacterial abundance and diversity in the marine environment (Breitbart, 2012). This is also true for the marine fish hatchery environment. Viral infections of bacterial hosts may lead to bacterial lysis and hence reduction of the bacterial population with a simultaneous creation of lysate particles which are essentially nutrients used by other bacteria to grow. In addition to the direct effects of exclusively lytic phages, temperate phages may become integrated in the bacterial chromosomes transferring new attributes like virulence factors or antibiotic resistant determinants to their hosts with a process known as lysogenic conversion. Bacteria and phages are in a constant “arms race” in the marine environment which drives evolution for both parties (Kalatzis et al., 2018). Furthermore, phage attack may lead to biofilm or aggregate formation as a means of defense for many bacterial species (Hansen et al., 2019). Phages can also interfere with the QS of the bacterial host which regulates biofilm formation (Hansen et al., 2019). These types of interaction may also be relevant for the members of *Tenacibaculum* genus since an interplay between planktonic and biofilm state has been observed in several marine hatcheries and may contribute to their pathogenicity but also to their overall ecological role.

To our knowledge, very few phages infecting *Tenacibaculum* spp. have been isolated and only one, has been genetically characterized, the lytic phage Ptm1, infecting the fish pathogenic bacterium *T. maritimum* (Kawato et al., 2020). In this paper, we describe the isolation and characterization of a novel *Tenacibaculum* phage-host system which may become useful for the study of the role of these bacteria in the marine hatcheries.

## 2. Materials and methods

### 2.1. Bacterial analysis

#### 2.1.1. Bacterial strains

The bacterium, designated as strain LAR25 was isolated in Marine Agar (MA) supplemented with mannitol from the inlet water of a commercial gilthead seabream and European seabass hatchery, located in Larymna, Central Greece. LAR25 was identified as *Tenacibaculum* sp. using molecular methods (PCR amplifying 16S rRNA) and whole genome sequencing. Additionally, five different bacterial strains belonging to four *Tenacibaculum* species (*T. discolor*, *T. mesophillum*, *T. soleae*, and *T. galaicum*) kindly offered by Dr. Mikkel Bentzon-Tilia of the Department of Biotechnology and Biomedicine of the Technical University of Denmark were used in the analysis. All the bacterial strains were maintained in Microbank tubes (Pro-lab Diagnostics, Richmond Hill, Canada) at −80°C and were grown in Lysogeny Broth (LB) (10 gL^−1^ tryptone, 5 gL^−1^ yeast extract, 10 gL^−1^ NaCl, 0.75 gL^−1^ MgSO_4_, 1.5 gL^−1^ KCl, 0.73 gL^−1^ CaCl_2_) at 25°C when used.

#### 2.1.2. Phenotypic characteristics

Cell morphology of LAR25 was studied with light and transmission electron microscopy (TEM) at the Electron Microscopy Laboratory of the University of Crete. Bacterial cells obtained from an overnight bacterial culture in LB broth, were harvested and following centrifugation at 13,000 × g for 3 min and reconstitution in saline (0.9% NaCl) were placed in microscopy slides and photographed alive using a Nikon 50i Eclipse microscope equipped with a Nikon digital microscopy camera. For TEM observation an aliquot of LAR25 was negatively stained with 4% w/v uranyl acetate (pH 7.2). Motility was observed under light microscope and by inoculating bacteria in Motility Indole Ornithine Medium (MIO, Sigma Aldrich Co., St. Louis, MO, United States).

#### 2.1.3. Salinity tolerance

The tolerance of LAR25 in different salinities was tested using LB medium broth which was prepared with filtered seawater and artificial seawater (23.4 gL^−1^ NaCl, 24.7 gL^−1^ MgSO_4_ ×7H_2_O, 1.5 gL^−1^ KCl and 1.43 gL^−1^ CaCl_2_ ×2H_2_O). Both LB medium broths (natural and artificial seawater) were adjusted to different salinities (39, 26, 19.5, 13, 9.7, 6.5, and 3.9‰). Growth of LAR25 was tested using the Infinite PRO 200 microplate reader (Tecan Trading AG, Switzerland) which was equipped with temperature control. Briefly, 200 μL of each LB broth with different salinities were loaded in triplicates in a sterile 96-well plate and inoculated with 20 μL of an overnight culture of LAR25. The plate was placed in the reader and incubated at 25°C while OD600 measurements were recorded every 10 min for 18 h.

#### 2.1.4. Catalase/oxidase/indole and flexirubin test

The bacterium LAR25 was tested for catalase production by the standard H_2_O_2_ method, for oxidase activity using disks (Honeywell International Inc., Charlotte, NC, United States), for the detection of flexirubin type pigments with 20% KOH and for the decomposition of tryptophane to indole, using Kovac’s reagent for indole (Sigma-Aldrich, Co., St. Louis, MO, United States).

#### 2.1.5. Antibiotic sensitivity

Bacterial suspension of LAR25 was diluted to obtain a 0.7 absorbance read at OD600 and plated on Mueller-Hinton agar (Difco, Detroit, MI, United States) supplemented with 2% NaCl. Antimicrobial susceptibility disks (Oxoid, Thermo Fisher Scientific Inc., Waltham, MA, United States) were placed on the agar plates and incubated at 25°C. The inhibition diameter was recorded after incubation for 24 h. The antibiotics tested were Tetracycline TE30, Flumequine UB30, Oxolinic acid OA2, Piperacillin PRL100, Sulfamethoxazole/Trimethoprim SXT25, Florfenicol FFC30 and Oxytetracycline OT30.

#### 2.1.6. Fatty acid analysis

An active growing culture of LAR25 on LB agar plate was sent to the Leibniz Institute DSMZ-German Collection of Microorganisms and Cell Cultures GmbH where they performed the cellular fatty acids analysis. Briefly, analysis was done following conversion into fatty acid methyl esters (FAMEs) by saponification, methylation and extraction using minor modifications of previously published methods (Miller, 1982; Kuykendall et al., 1988). The fatty acid methyl esters mixtures were separated by gas chromatography and detected by a flame ionization detector using Sherlock Microbial Identification System (MIS) (MIDI, Microbial ID, Newark, DE, United States). Peaks were automatically integrated, and fatty acid names and percentages calculated by the MIS Standard Software (Microbial ID). In subsequent analysis, summed features were resolved and identities of fatty acids were confirmed by a GC–MS-based analysis using retention time locking and mass spectral data.

#### 2.1.7. Bacterial genomics

DNA was extracted from overnight cultures (LB broth, 25°C) using the DNeasy Blood and Tissue kit (QIAGEN, Hilden, Germany). The concentration was measured with NanoDrop (Thermo Fisher Scientific Inc., Waltham, MA, United States) and Qubit (Thermo Fisher Scientific, Waltham, MA, United States) instruments and the sample integrity was assessed with 1% agarose gel electrophoresis. The whole genome was sequenced by the DNBseq platform (BGI Tech Solutions, Hong Kong) on the DNBSEQ-G400 sequencer using paired-end technology (PE100). The library preparation workflow for the platform consisted of fragment selection, end repair and A-tailing, bubble adaptor ligation, PCR amplification, splint circularization, digestion and purification and DNB making. Raw reads were filtered if more than 25% matched the adapter sequence, if more than 50% bases had quality values lower than 20 and if there were more than 3% N in the read. Filtering was completed using the SOAPnuke software (Chen et al., 2018). After filtering, the clean reads were uploaded in PATRIC, Pathosystems Resource Integration Center (Davis et al., 2019), that provides analysis tools to support biomedical research on bacterial infectious diseases. Then, the assembly process involved Samtools 1.3 (Danecek et al., 2021), the Unicycler v0.4.8 (Wick et al., 2017) assembler, Pilon 1.23 (Walker et al., 2014) correction and Bandage 0.8.1 (Wick et al., 2015) visualization. The clean reads were also uploaded to the web based PLACNETw (Vielva et al., 2017), in order putative plasmids to be reconstructed and visualized. The contigs were submitted and annotated to the GenBank, NCBI database, with PGAP (Li et al., 2021) accession number JAIWJY000000000, the clean reads were also submitted to the Sequence Read Archive (SRA) database in BioProject PRJNA765488. Seventeen WGS from more than 10 species were retrieved from NCBI Reference Sequence. The group consisted of 1 strain *T. caenipelagi* (CECT 8283), 1 strain of *T. dicentrarchi* (AY7486TD), 2 strains of *T. discolor* (DSM 18842, IMLK18), 1 strain of *T. gallaicum* (DSM 18841), 1 strain of *T. jejuense* (KCTC 22618), 1 strain of *T. litoreum* (HSC 22), 1 strain of *T. lutimaris* (DSM 16505), 2 strains of *T. maritimum* (TM-KORJJ, NCIMB 2154T), 1 strain of *T. mesophilum* (DSM 13764), 1 strain of *T. singaporense* (DSM 106434), 4 strains *Tenacibaculum* sp. (AHE15PA, AHE14PA, SZ-18, 4G03), 1 strain of *T. todarodis* (LPB0136). Supplementary Table S1 provides the information of the genomes included in this study.

#### 2.1.8. Phylogenetic analysis

Genome-wide comparisons were performed, a heatmap based on OrthoANI values was generated by Orthologous Average Nucleotide Identity Tool (OAT) software (Lee et al., 2016) and genome similarity ANI-matrix was calculated with the genome-based distance matrix calculator (Rodriguez-R and Konstantinidis, 2016). The multi-locus sequence typing (MLST) was used to characterize and set a sequence type for the LAR25 strain, isolates’ MLST profiles were downloaded from the Public Databases for Molecular Typing and Microbial Genome Diversity (Jolley et al., 2018) and a phylogenetic tree based on the allelic schemes was constructed with the Neighbor-joining method, using Geneious v.9.1.6 (Biomatters Ltd., Auckland, New Zealand). The LAR25 strain with its assigned alleles were also submitted to the PubMLST database (isolate ID: 154).

#### 2.1.9. Functional annotation of the novel bacterium

Annotation and functional categorizing were gathered by the BlastKOALA tool of the Kyoto Encyclopedia of Genes and Genomes (KEGG) database (Kanehisa et al., 2016) as well as by manual curation. Special gene predictions involving secondary metabolites, carbohydrate-active enzymes (CAZymes), antiphage systems were delivered by the antiSMASH 6.1.1 (Blin et al., 2021), the dbCAN2 (Zhang et al., 2018), and the DefenseFinder (Abby et al., 2014; Tesson et al., 2022) webservers, respectively. The dbCAN2 meta server employed for the automated annotations HMMER search against dbCAN HMM, and dbCAN-sub databases, with e-value <1e^−15^ and coverage >0.35 and the DIAMOND against CAZy pre-annotated CAZyme sequence database with cutoff e-value <1e^−102^. The hits that were predicted with only one tool and with coverage <0.8 were filtered out to improve accuracy. The CAZyme gene clusters (CGCs) that included transcription factors (TFs), transporters (TCs, STPs) were identified using CGC-Finder of the dbCAN2. Genomic islands (GIs) were predicted with the webtool IslandViewer v4 (Bertelli et al., 2017), using two independent methods SIGI-HMM and IslandPath-DIMOB. The resulting GIs that were not located in aligned areas against reference genome *Tenacibaculum* sp. LPB0136, were filtered out. Prophages were identified using the PHASTER webserver (Arndt et al., 2016). The IntegronFinder 2.0 (Néron et al., 2022), as implemented in the Galaxy/Pasteur webserver (Afgan et al., 2018), was used to search for integron elements.

### 2.2. Bacteriophage isolation and characterization

#### 2.2.1. Phage isolation and propagation

Water samples were collected from the outlet of a commercial fish hatchery located in Larymna, Greece. The phage was isolated using standard methodology described previously (Misol et al., 2020). Briefly, 500 mL of the collected water were enriched with 50 mL of concentrated LB (10×) and inoculated with 5 mL of the host strain, *Tenacibaculum* sp. strain LAR25 overnight liquid culture. The enriched water sample was incubated at 25°C with a shaking speed of 100 rpm for 24 h. Following centrifugation at 13,000 × *g* for 3 min, the supernatant was filtered through a 0.22 μm sterile filter (GVS Life Sciences, Sanford, ME, United States) and 10 μL aliquots were tested for clearing zones on bacterial lawns of the host strain. Following 24 h incubation at 25°C, the clearest plaques were collected. Isolated plaques were picked and purified by replating five times with the double-layer agar method to ensure clonal phage stocks. The purified phage was propagated with the double-layer agar method in high titer (10^12^ pfu mL^−1^), stored at 4°C for further characterization and was named Tenacibaculum phage Larrie.

#### 2.2.2. Phage morphology

Phage aliquot with a titer of ~10^11^ pfu mL^−1^ was prepared for TEM observation and negatively stained with 4% w/v uranyl acetate (pH 7.2). The phage was observed using a JEOL transmission electron microscopy operated at 80 kV at the Electron Microscopy Laboratory in the University of Crete. From the obtained digital micrographs, capsid width and tail length of individual phages (*n* = 20) were measured with ImageJ software version 1.53p.

#### 2.2.3. Host range and efficiency of plating (EOP)

The determination of the host range and the efficiency of plating (EOP) of the phage Larrie was performed according to Kutter (2009). Briefly, bacterial strains were cultured in LB broth at approximate concentration of 10^7^ cfu mL^−1^, mixed with top molten LB agar and poured on bottom LB agar. Following solidification of top agar, 10 μL of serial diluted phage were spotted on the different bacterial lawns. The phage titer was determined after incubation of the plates at 25°C for 24 h.

#### 2.2.4. Thermal and pH stability

For temperature sensitivity assessment, aliquots (1 mL) of Larrie phage (10^12^ pfu mL^−1^) were incubated for 1 h at 25, 35, 40, 45, 50, 55, 65, and 75°C, respectively. Phage stored at 4°C was used as a control. For pH stability test, LB medium was adjusted with 1 M NaOH or HCL to achieve a pH range 1–10 and phage aliquots (1 mL) were suspended in each of them following incubation for 1 h at 25°C. For both assays, phage titers were analyzed using the double-layer agar method after incubation of the plates for 24 h at 25°C. The experiments were repeated three times.

#### 2.2.5. One-step growth

For one step growth, Tenacibaculum phage Larrie was added to 1 mL of host bacterial culture in early exponential phase (OD_600_ = 0.2) at MOI = 0.01 and incubated for 15 min at 25°C. Following centrifugation at 13,000 × *g* for 3 min, the supernatant containing free phages was discarded while phages that managed to attach to the bacteria were pelleted on the bottom of the tube. Pellet was suspended in 1 mL and then transferred in 19 mL fresh liquid medium. This moment was considered as *t* = 0 and 1 mL was collected from the infected host culture, serially diluted and 10 μL was spotted on petri dishes containing bacterial lawn of the host. This step was repeated at 10 min intervals for total duration of 100 min. Phage plaques were counted after incubation for 24 h at 25°C.

#### 2.2.6. *In vitro* cell lysis

The *in vitro* cell lysis assay was performed using the Infinite PRO 200 microplate reader (Tecan Trading AG, Switzerland) equipped with temperature control. For the assay, 200 μL of freshly prepared culture of the host bacteria were loaded in a sterile 96-well plate, placed in the reader and incubated at 25°C. When the bacteria cultures were at the exponential phase (~10^8^ CFU mL^−1^) they were infected with the phage Larrie at 3 different MOIs (0.1, 1 and 10 in triplicates). Three wells were not infected with phage and served as control. The plate was placed in the reader and OD_600_ measurements were recorded every 10 min for 24 h.

#### 2.2.7. Statistical analysis

One-Way Analysis of Variance (ANOVA) was performed to test any differences in the titer of the phage at the thermal and pH stability assays, followed by Tukey’s post-hoc test in cases where significant differences were found with ANOVA (*p* < 0.05), calculated in GraphPad Prism version 9.1.

#### 2.2.8. Phage genomic analysis

*Tenacibaculum* phage Larrie’s sequence reads were assembled by Unicycler v0.4.8 (Wick et al., 2017) in PATRIC (Davis et al., 2019). The quality of assembly was assessed by QUAST v4.6.3 (Gurevich et al., 2013). The RASTtk (Brettin et al., 2015) and Glimmer (Delcher et al., 2007) were used for gene prediction and the predicted genes were manually inspected to ensure the presence of valid phage start codons (ATG/GTG or TTG). Furthermore, (i) NCBI Basic Local Alignment Search Tool (BLAST) (Altschul et al., 1990; Camacho et al., 2009) adjusted at non-redundant (nr) protein database, (ii) Gene Ontology (Gene Ontology Consortium, 2004), and (iii) InterPro (Mitchell et al., 2019), were used for the functional annotation of the predicted genes. TΜHMM 2.0 (Krogh et al., 2001) was used for the prediction of possible transmembrane proteins. NCBI Conserved Domain Database (NCBI CDD) (Lu et al., 2020) was used to detect conserved domains within the protein-coding regions. The presence of integrase, virulence and antibiotic resistance-encoding genes was examined through INTEGRALL Database webserver (Moura et al., 2009), Virulence Factor Database (VFDB) (Chen, 2004), VirulenceFinder and ResFinder webservers (Kleinheinz et al., 2014). Possible tRNAs were predicted using ARAGORN (Laslett, 2004) through Galaxy webserver (Afgan et al., 2018). BACPHLIP was used to predict the phage lifestyle (Hockenberry and Wilke, 2021). PhageTerm analysis through the Galaxy webserver was conducted in order to predict genome ends and DNA packaging mechanism. Only probabilities above 90% were accepted for manual protein function assignment of the Larrie’s predicted ORFs. All hits in existing databases were expected to have E-value below 10^−3^. Genome circular representation was conducted with Geneious v.9.1.6 (Biomatters Ltd., Auckland, New Zealand)^1^ and CGview.^2^

#### 2.2.9. Genome alignment and phylogenetic analysis

ViPTree (Nishimura et al., 2017) was used to compare Larrie’s proteome to 4,982 dsDNA phage proteomes to construct a viral proteomic tree. Moreover, the genomes of other *Tenacibaculum* phages were downloaded from NCBI VIRUS database (Hatcher et al., 2017) and were aligned with Larrie’s genome using progressiveMauve: Multiple alignment to assess genomic synteny (Darling et al., 2010). Large terminase subunits of referred phages and Larrie were downloaded and were aligned using MUSCLE algorithm (Edgar, 2004). Using Molecular Evolutionary Genetic Analysis (Mega X) (Kumar et al., 2018), a maximum likelihood phylogenetic tree was constructed based on large terminase subunuits, using Tamura-Nei 93 model with bootstrap test = 1,000. Gaps in the amino acid sequence alignments were trimmed. The tree was visualized using Interactive Tree of Life web server (Letunic and Bork, 2021).

#### 2.2.10. Statistical analysis

One-Way Analysis of Variance (ANOVA) was performed to test any differences in the titer of the phage at the thermal and pH stability assays, followed by Tukey’s post-hoc test in cases where significant differences were found with ANOVA (*p* < 0.05), calculated in GraphPad Prism version 9.1.

## 3. Results

### 3.1. Bacterial analysis

#### 3.1.1. Bacterial morphology

**Figure 1:**
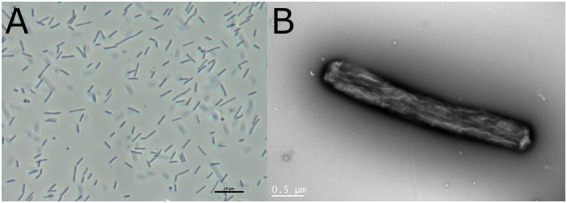
Cell morphology of *Tenacibaculum* sp. LAR25 as observed by light and electron microscopy. **(A)** Phase-contrast micrograph of fresh culture of the bacteria in LB broth showing both elongated and spherical cells. **(B)** Negatively stained cell of the novel bacterium observed with TEM.

Cells were aerobic, Gram-negative, non-flagellated, non-spore- forming, straight rods (0.3–0.4 μm in width and 3.0–3.3 μm in length) that were non-motile (Figure 1). Spherical cells were occasionally observed. The bacterium formed yellowish round shaped colonies on LB agar plates.

#### 3.1.2. Salinity tolerance

**Figure 2:**
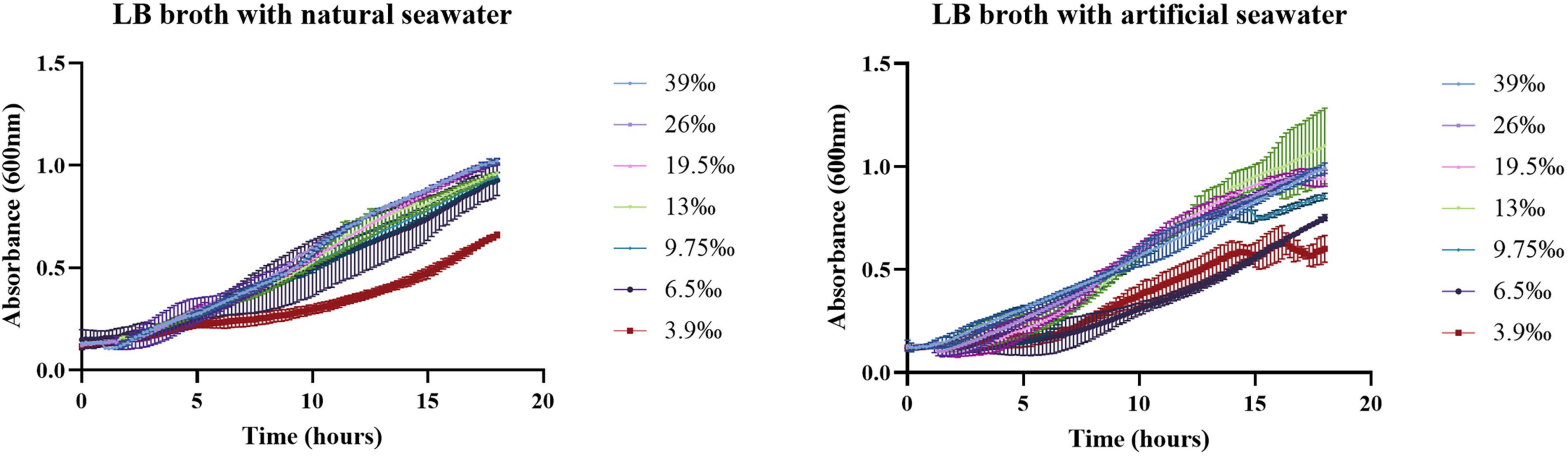
Growth of LAR25 in LB medium broth with natural seawater and artificial seawater adjusted to different salinities. Values are mean ± SD (*n* = 3).

No differences were observed in the growth of LAR25 in LB with natural seawater at a range of salinities from 6.5–39‰ while growth reduction was observed after 18 h of incubation at 3.9‰. In the LB with artificial seawater a similar pattern was observed but in this case reduction was observed at 9.75, 6.5, and 3.9‰ (Figure 2).

#### 3.1.3. Antibiotic sensitivity

The results of the antibiotic sensitivity of LAR25 are presented in Table 1. LAR25 appeared sensitive to Flumequine, Sulfamethoxazole/Trimethoprim and Florfenicol and resistant to Oxolinic acid.

**Table 1:** Antibiotic susceptibility of *Tenacibaculum* sp. LAR 25.

Antibiotic	Diameter (cm)	Sensitivity
O129	1.7	
Tetracycline (TE30)	1.5	I
Flumequine (UB30)	1.6	S
Oxolinic acid (OA2)	-	R
Piperacillin (PRL100)	1.9	I
Sulfamethoxazole/trimethoprim (SXT25)	2.7	S
Florfenicol (FFC30)	3.3	S
Oxytetracycline (OT30)	1.5	I

#### 3.1.4. Fatty acid profile

The profile of the cellular fatty acids of the strain LAR25 is presented in Table 2. The major fatty acids were iso-C15:0, iso-C15:1 ω10c, iso-C15:0 3-OH, iso-C17:0 3-OH and summed feature 3 comprised of iso-C15:0 2OH and 16:1 ω7c.

**Table 2:** Cellular fatty acid composition (%) of strain LAR25.

Fatty acid	Tenacibaculum LAR25	T. soleae	T. mesophilum	T. lumimaris	T. skagerrakense	T. maritimum	T. litoreum	T. aestuarii	T. litopenaei	T. discolor	T. maritimum
**Straight chain**											
C15: 0		4.8	3.6	8.9	4.9	2.9	2.7	6.1		3	1.4
C16: 0	0.62	tr	tr	tr	tr	tr	tr	tr	1.8		
**Branched chain**											
iso-C13: 0	0.52	tr	tr	tr	tr	1.8	1.4	1.3	tr	tr	1.3
iso-C15: 0	19.3	23	13	17	9.5	17	19	19	22	18	21
anteiso-C15: 0	1.61	tr	1.1	tr	–	tr	1.8	2	tr	1.1	1.3
iso-C15: 1		5.7	7.1	5.3	8.2	7.6	8.2	8.7	8.7		
iso-C15: 1G (*GC MS: iso-C15: 1 ω10c*)	7.15									8.1	12
iso-C16: 0	2.32	1.7	1.7	3.8	1.3	tr	2.3	2.3	1.8	3.3	tr
iso-C16: 1 H (*GC MS: iso-C16: 1 ω6c*)	2.26									2.7	1.9
**Unsaturated**											
C15: 1ω6c	2.58	12	1.6	4.2	–	2.2	1.7	3	1.6	3.2	3.9
C17: 1ω6c	1.57	1.7	tr	1.5	1.2	tr	tr	1.6	1.9	1.7	tr
**Hydroxylated**											
iso-C15: 0 3-OH	7.07	11	8	4.6	7.8	20	6.6	6.1	4.6	5.2	15
C15: 0 3-OH	3.27	3.2	2.9	3.4	8.6	3.8	–	4.2	2.7	2.3	1.4
iso-C16: 0 3-OH	10.3	8.4	9	13	12	5	6.8	12	3.4	12	5.9
C16: 0 3-OH	3.08	2.2	3.2	1.3	2.1	1.5	1.6	1	5.4	1.4	1.2
iso-C17: 0 3-OH	11.3	2.9	15	8.4	12	14	14	9.6	13	9.1	8.5
Summed features*											
Summed feature 3	20.3	11	24	18	23	18	20	12	21	18	14
Summed feature 9	1.22										

Only fatty acids amounting >0.5% of the total are shown. The fatty acid profiles of congeneric species are shown for comparison and were analyzed elsewhere (Piñeiro-Vidal et al., 2008a,b). tr: traces.

#### 3.1.5. Genomic features of LAR25 and phylogeny

Genome assembly resulted in 50 contigs with a total size is 3,660,580 bps with 31.80% G + C content. The *Tenacibaculum* sp. LAR25 genomic features are listed in Table 3. It contains 3,395 predicted genes, of which 3,339 are coding sequences (CDSs). No plasmids were detected *in silico*. There were 3 incomplete predicted prophage regions of 7.4, 5.8, and 7.2 kb in size (Supplementary Table S2). They incorporated approximately 8 coding sequencies each, including baseplate subunits, chaperonins, diamenases, DNA polymerases, metallophosphoesterases. PHASTER analysis revealed similarity of LAR25 proteins with proteins of phages such as Tenacibaculum phage PTm1, Flavobacterium phage vB_FspM_lotta8-1 and Bordetella phage vB_BbrM_PHB04. Whereas the blast search of the regions showed similarity with regions of other *Tenacibaculum* spp. genomes (data not shown). There were 9 GIs in LAR25 around 30 kbps, constituting only 0.8% of the genome and there were not any integrons present.

**Table 3:** General genomic characteristics of *Tenacibaculum* sp. strain LAR25.

Accession number	JAIWJY000000000
Length (bp)	3,660,580
G + C (%)	31.83
Contigs	50
N50	404,126
Genes	3,395
CDS	3,339
5S rRNAs	1
16S rRNAs	1
23S rRNAs	1
Genes (RNA)	56
rRNAs	3
complete rRNAs	3
tRNAs	49
ncRNAs	4
Pseudo Genes (total)	19

**Figure 3:**
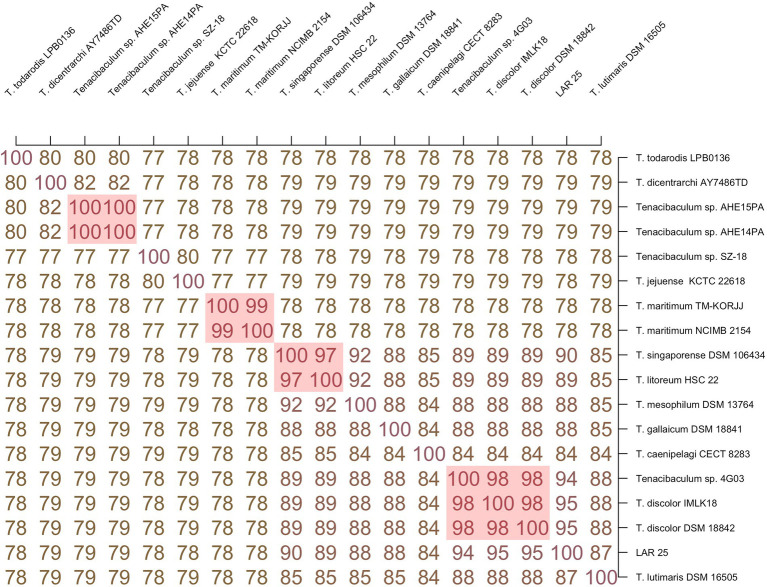
Genome similarity ANI-Matrix of LAR25 and 17 genomes *Tenacibaculum* spp.

**Figure 4:**
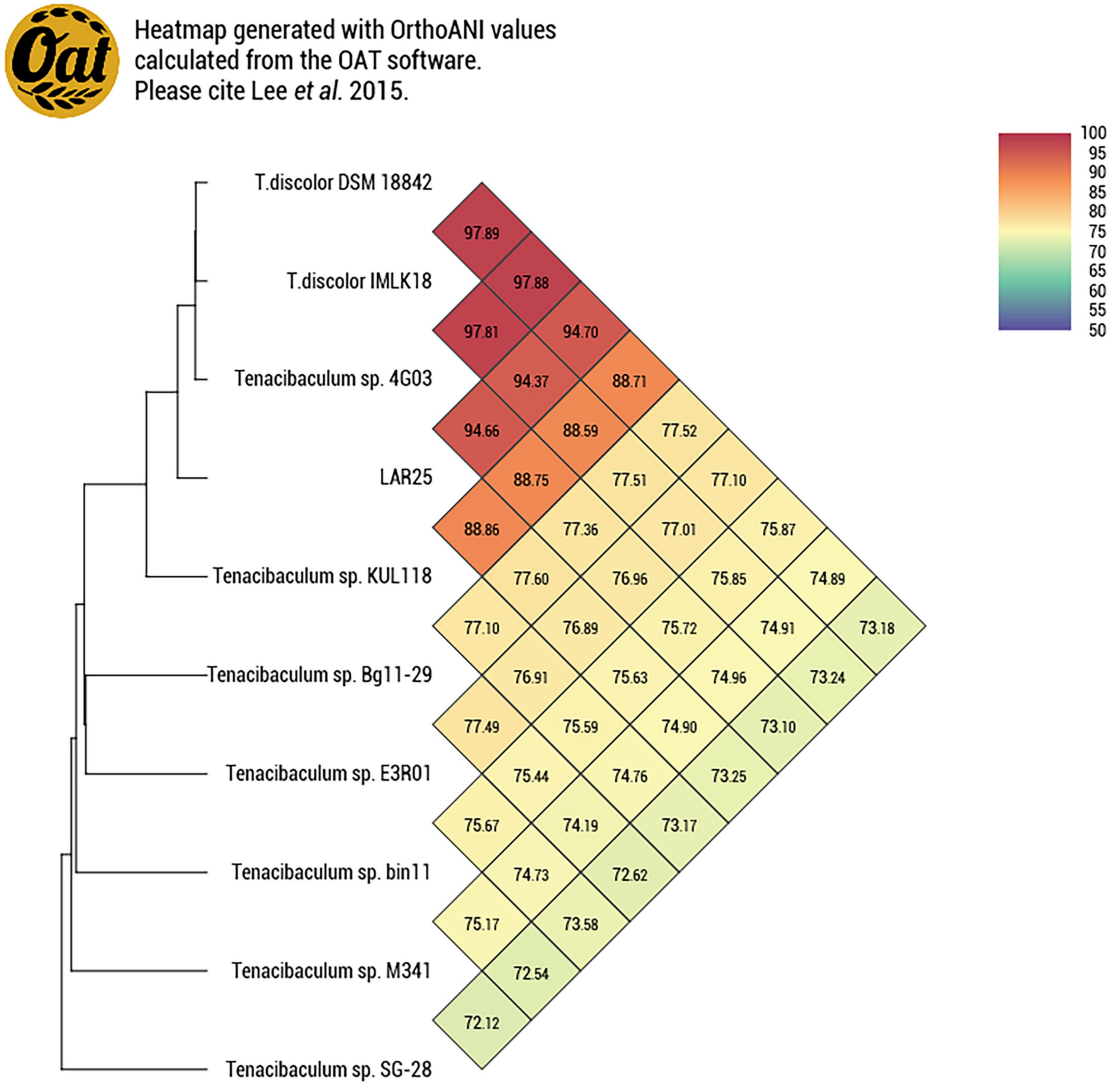
Heatmap with OrthoANI values for the LAR25 strain and 9 genomes of *Tenacibaculum* spp. [Heatmap generated with OrthoANI values calculated from the Orthologous Average Nucleotide Identity Tool (OAT) software].

**Figure 5:**
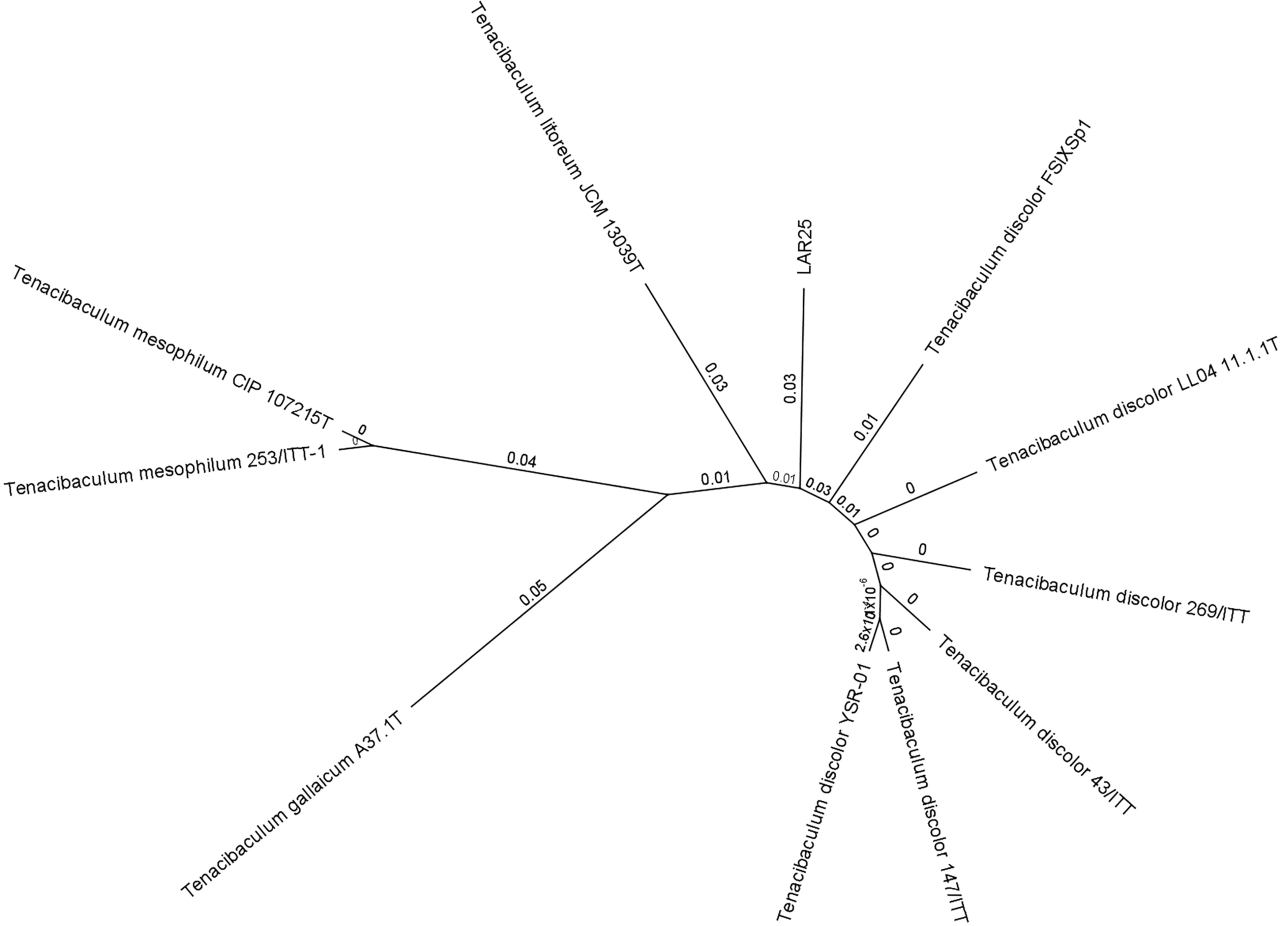
Phylogenetic relationships between *Tenacibaculum* strains based on neighbor joining (NJ)-multilocus sequence typing (MLST) analysis on concatenated sequences of *atpA, dnaK, glyA, gyrB, infB, rlmN, tgt* genes. Labels on branches indicate substitution per site values.

ANI analysis was performed to estimate genomic differences and relatedness between the LAR25 and 17 genomes of the genus *Tenacibaculum* (Figure 3). Apparently, LAR25 is closer to *T. discolor*. The genomes of *T. discolor* strains together with the *Tenacibaculum* sp. strain 4GO3 shared ANI values ranging from 94 to 95% (highest similarity was with *T. discolor* at 94.56%), which are values at the threshold of the speciation boundary of 95% identity. Further genome-similarity analysis with OrthoANI (Figure 4), confirmed that the highest similarity of LAR 25 was with *T. discolor* DSM 18842 at 94.7%. LAR25 diverged from other *Tenacibaculum* spp. The phylogenetic relationships of LAR25 and *Tenacibaculum* sequences deposited in the PubMLST database corroborated also that it is closely related to *T. discolor* strains isolated from European seabass (Supplementary Table S3) from Italy (Figure 5).

#### 3.1.6. Niche adaptation arsenal- metabolism, virulence, resistance

Important features of metabolic pathways, virulence and antimicrobial resistance are presented in Table 4. Detailed results of the locus tags and the KEGG annotated features are presented in Supplementary Table S4. The strain LAR25 possesses genes of various virulence factors involving secretion systems, transporters, siderophores, pili, extracellular proteins, quorum sensing, prokaryotic defense, antimicrobial resistance, metabolism of carbohydrates, secondary metabolites, polyketides and terpenoids.

**Table 4:** Genes related to putative virulence factors, stress response and metabolism elements present in the LAR25 genome.

Category	Feature		Gene count
**Membrane transport**			
	ABC transporters		18
	Bacterial secretion system	T1SS	1
		T2SS	3
		T3SS	1
		T4SS	1
		T6SS	7
		T9SS	21
		Curli production assembly	2
		Sec-SRP system	9
		Tat system	3
**Cell motility**			
	Bacterial chemotaxis	*ompA/motB*	2
	Pilus system		4
**Cellular community**			
	Quorum sensing		27
		MBL fold metallo-hydrolase	11
		Transcriptional response regulator OmpR family protein	2
		LytTR family DNA-binding domain-containing protein	1
	Quorum quenching	*ahlD*	1
	Biofilm formation		9
**Extracellular proteins**			
		RIP metalloprotease rseP	1
		Zinc-dependent metalloprotease	1
		zinc metallopeptidase	1
		Bifunctional metallophosphatase/5′-nucleotidase	1
		Ceramidase	1
		*prtC*	1
	Bacterial toxins	Hemolysin	2
		Hemolysin III	1
		Cholesterol-dependent/thiol-activated cytolysin	1
		Magnesium and cobalt exporter	2
**Stress response**			
	Toxin-Antitoxin system (TA system)		5
	Osmotic stress response	*cls*	1
	Oxidative stress response	*sodA*	1
	ROS detoxification	*katG*	1
	Antiviral system	*drt4*	1
		*ptuB,ptuA*	2
		*avs2A*	1
		*qatA,qatB,qatC,qatD*	4
		RT 1 C3	1
		Phospholipase,E2,Cyclase II	3
		*tmnA*	1
		*gajB 1,gajA*	2
		Type IIG	1
		Type I MTases,Type I S,Type I REases	3
		*hamB,hamA1*	2
		Mokosh TypeII mkoC	1
**Iron acquisition**			
	Ferrous iron transport	MFS efflux protein	1
		*feoA*	1
		*feoB*	1
	Iron source	ferritin	2
	Siderophore biosynthesis		1
	Heme uptake	TonB-dependent receptor	32
**Lipid metabolism**			
	Fatty acid biosynthesis		18
	Fatty acid degradation		11
	Glycerolipid metabolism		6
	Glycerophospholipid metabolism		10
	Arachidonic acid metabolism		1
	Linoleic acid metabolism		1
	Alpha-Linolenic acid metabolism		1
	Biosynthesis of unsaturated fatty acids		2
**Metabolism of cofactors and vitamins**			
	Thiamine metabolism		13
	Riboflavin metabolism		6
	Vitamin B6 metabolism		4
	Nicotinate and nicotinamide metabolism		13
	Pantothenate and CoA biosynthesis		19
	Biotin metabolism		10
	Lipoic acid metabolism		2
	Folate biosynthesis		18
	One carbon pool by folate		13
	Porphyrin metabolism		21
	Ubiquinone and other terpenoid-quinone biosynthesis		10
**Metabolism of terpenoids and polyketides**			
	Terpenoid backbone biosynthesis		9
	Carotenoid biosynthesis		3
	Zeatin biosynthesis		1
	Limonene and pinene degradation		3
	Geraniol degradation		3
	Biosynthesis of ansamycins		2
	Biosynthesis of type II polyketide products		1
	Polyketide sugar unit biosynthesis		5
	Nonribosomal peptide structures		1
	Biosynthesis of siderophore group nonribosomal peptides		1
	Biosynthesis of vancomycin group antibiotics		1
**Xenobiotics biodegradation and metabolism**			
	Benzoate degradation		10
	Aminobenzoate degradation		2
	Chloroalkane and chloroalkene degradation		2
	Xylene degradation		2
	Ethylbenzene degradation		1
	Styrene degradation		3
	Caprolactam degradation		2
	Dioxin degradation		1
	Polycyclic aromatic hydrocarbon degradation		1
**Antimicrobial resistance**			
	beta-Lactam resistance	*oprM*	2
		*blaB*	1
		*tolC*	1
		*mrdA*	1
		*mrcA*	2
		*ftsI*	1
		*mexA*	3
		*blaI*	2
		*nagZ*	1
	Vancomycin resistance	*vanX*	1
		*murG,F*	2
		*ddl*	1
		*alr*	2
		*mraY*	1
	Cationic antimicrobial peptide (CAMP) resistance	*lpxA*	1
		*amiABC*	2
		*PPIA*	2
		*degP/htrA*	1
	Phenicol resistance	*catA*	1
	Tetracycline resistance	*tetM/O*	1

There were parts of 9 types of secretion systems including T2SS, T6SS, T9SS, and Sec-SRP. Among the quorum sensing genes, there was also the quorum quenching gene N-acylhomoserine lactonase (*ahlD*). Extracellular and secreted proteins predicted for LAR25 are proteases, the ceramidase, the collagenase gene *prt*, the magnesium/cobalt transporters *corA* and *mgtE*, and few toxins as well. LAR25 contains mechanisms for iron uptake, transport and storage, although ferritin can also be involved in the oxidative stress response. Other stress response related genes are the catalase *katG* and the osmotically regulated cardiolipin synthase *cls*. There were many genes related to beta-lactam, vancomycin, CAMP, phenicol and tetracycline resistance, although the phenotypic tests resulted in quinolone resistance.

The anti-phage systems found in the genome were of 12 types. Two restriction modification (RM) systems were among them. The CBASS II system had the oligonucleotide cyclase, the signaling molecule E2 encoded, along with the effector cell-killing gene, the phospholipase. Mokosh type II comprises the *mkoC*, that is an RNA helicase at the N-terminus and the PLD nuclease as the effector domain. A gene (reverse transcriptase) but not the whole retron system type I, has been identified, which can be a piece of a larger anti-phage defense system. The reverse transcriptase of DRT type 4 system was found, as well as an ATPase of the STAND superfamily of the AVAST type II, the QueC-like associated with ATPase and TatD DNases, nucleases and helicases from Gao Tmn/Qat, Hachiman, Gabija, and Septu systems.

The *Tenacibaculum* sp. strain LAR25 was able to produce, modify and degrade carbohydrate glycoconjugates, with 41 CAZymes predicted. There were 24 glycosyltransferases (GTs), 8 glycoside hydrolases (GHs), 4 carbohydrate esterases (CEs), an auxiliary activities enzyme (AA) and 4 carbohydrate-binding modules (CBMs) (Supplementary Table S5). The most common gene families detected were the synthases GT2, GT4. The 5 CGC1 polysaccharide utilization loci (PULs) had approximately 16 genes and 17.6 kbps length. The CAZymes of the clusters were the GH171, GH3, GT4, GH73, CBM50, AA1, GT2. The antiSMASH identified 5 secondary metabolite regions of average 42 kbps, with relaxed strictness. There were 6 types of gene clusters found, a NRPS (non-ribosomal peptide synthetase cluster) and a NRPS-like fragment, a bisucaberin B biosynthetic gene cluster from *Tenacibaculum mesophilum* (MIBiG repository: BGC0001531), a T1PKS (Type I Polyketide synthase), a terpene, and an Aryl polyene cluster. Other indicative genes of metabolic pathways of lipids, cofactors, vitamins and xenobiotics are presented in Table 4.

### 3.2. Description of *Tenacibaculum larymnensis* sp. nov.

*Tenacibaculum larymnensis* (la.rym. nen’sis. L. neut. adj. larymnensis from Larymna, a city of Central Greece, referring to the place of isolation).

The cells of *Tenacibaculum larymnensis* sp. nov. are aerobic, Gram-negative, non-flagellated, non-spore-forming, straight rods (0.3–0.4 μm in width and 3.0–3.3 μm in length) that are non-motile (Figure 1). Spherical cells are occasionally observed. On LB agar plates the bacterium forms yellowish round shaped colonies after 2 days incubation at 25°C. Growth occurs between 4°C to 45°C with optimum 25°C, between pH 6 to pH 8 with optimum pH 7 and between 3.9 to 39‰ salinity with optimum range between 13 to 39‰. Flexirubin-type pigments are not produced (KOH test negative). LAR25 is catalase-, oxidase-positive and negative for indole production. It is resistant to oxolinic acid, and sensitive to flumequine, sulfamethoxazole/trimethoprim and florfenicol. The major fatty acids were iso-C15:0, iso-C15:1 ω10c, iso-C15:0 3-OH, iso-C17:0 3-OH and summed feature 3 comprised of iso-C15:0 2OH and 16:1 ω7c. The DNA G + C content of the type strain is 31.81%. The type strain LAR25 was isolated from the inlet water of a fish hatchery located in Larymna, Greece and has been deposited to DSMZ bacterial collection under the accession number 11506.

### 3.3. Phage isolation and characterization

#### 3.3.1. Description of bacteriophage Larrie

**Figure 6:**
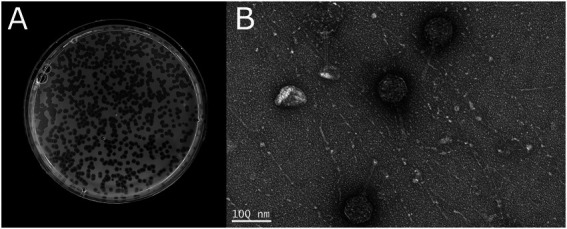
**(A)** Morphology of the plaques produced by the phage Larrie. **(B)** Phage particles negatively stained and observed with TEM.

Bacteriophage Larrie produced clear plaques (2.75 ± 0.79 mm) with perimetric halo on *Tenacibaculum larymnensis* sp. nov. strain LAR25 lawn (Figure 6A). The morphology of the phage as observed with TEM classifies Larrie as a siphovirus (Figure 6B). The phage had an icosahedral capsid 74.30 ± 2.67 nm (mean ± SD, *n* = 20) in diameter and a thin straight tail 98.64 ± 12.50 nm in length.

Larrie was able to lyse only its original host LAR25 and none of the other five bacterial strains belonging to four *Tenacibaculum* species (*T. discolor*, *T. mesophillum*, *T. soleae*, and *T. galaicum*) that were tested in this study.

**Figure 7:**
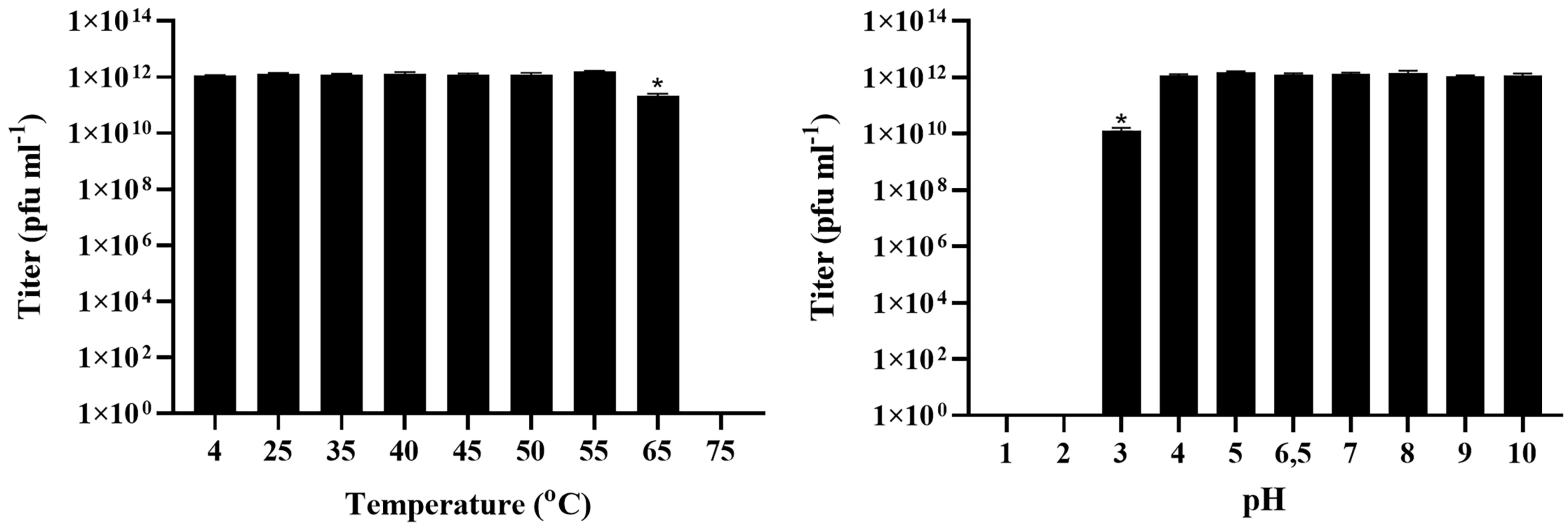
Effect of different temperatures and different pH on the stability of Tenacibaculum phage Larrie. Values are mean ± SD (*n* = 3) while (*) indicates statistically significant differences between different treatments (*p* < 0.05).

Temperature sensitivity assessment showed that the novel phage was stable between 4 and 55°C. At 65°C a statistically significant decrease of its titer was observed (*p* = 0.000), while from 75°C and above the phage was completed inactivated. The novel phage was stable from pH 4 to 10. A significant reduction of the titer was observed at pH 3 (*p* = 0.000) while pH 1 and 2 led to inactivation of the phage (Figure 7).

**Figure 8:**
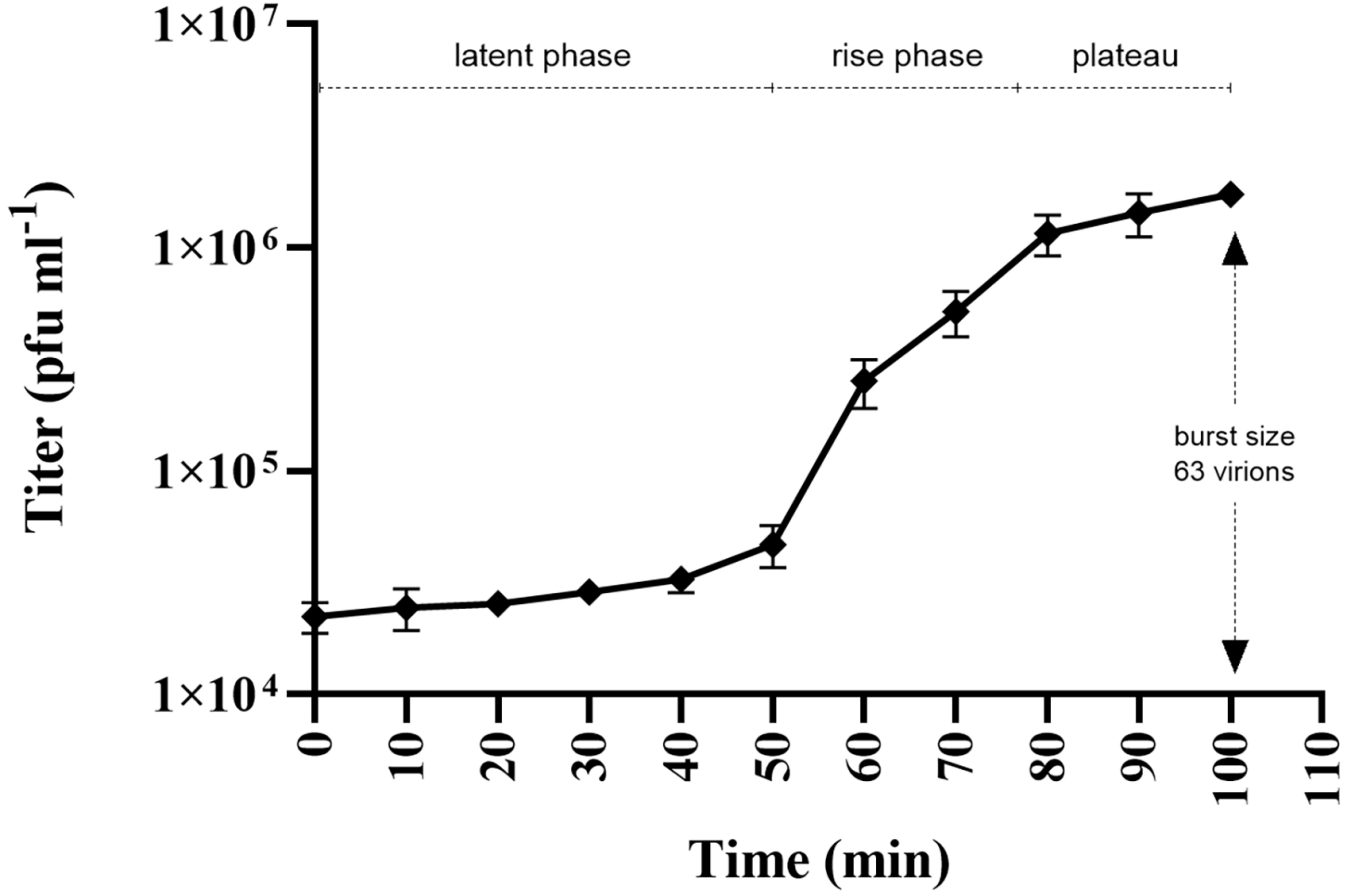
One-step growth curve of Tenacibaculum phage Larrie against host strain LAR25 at multiplicity of infection (MOI) 0.01. Values are mean ± SD (*n* = 3).

One-step growth assay showed that phage Larrie has a latent phase of 50 min. The rise phase was estimated between 50 and 80 min while the plateau phage was reached at 80 min. The burst size of phage Larrie was 63 virions (Figure 8).

#### 3.3.2. *In vitro* cell lysis

**Figure 9:**
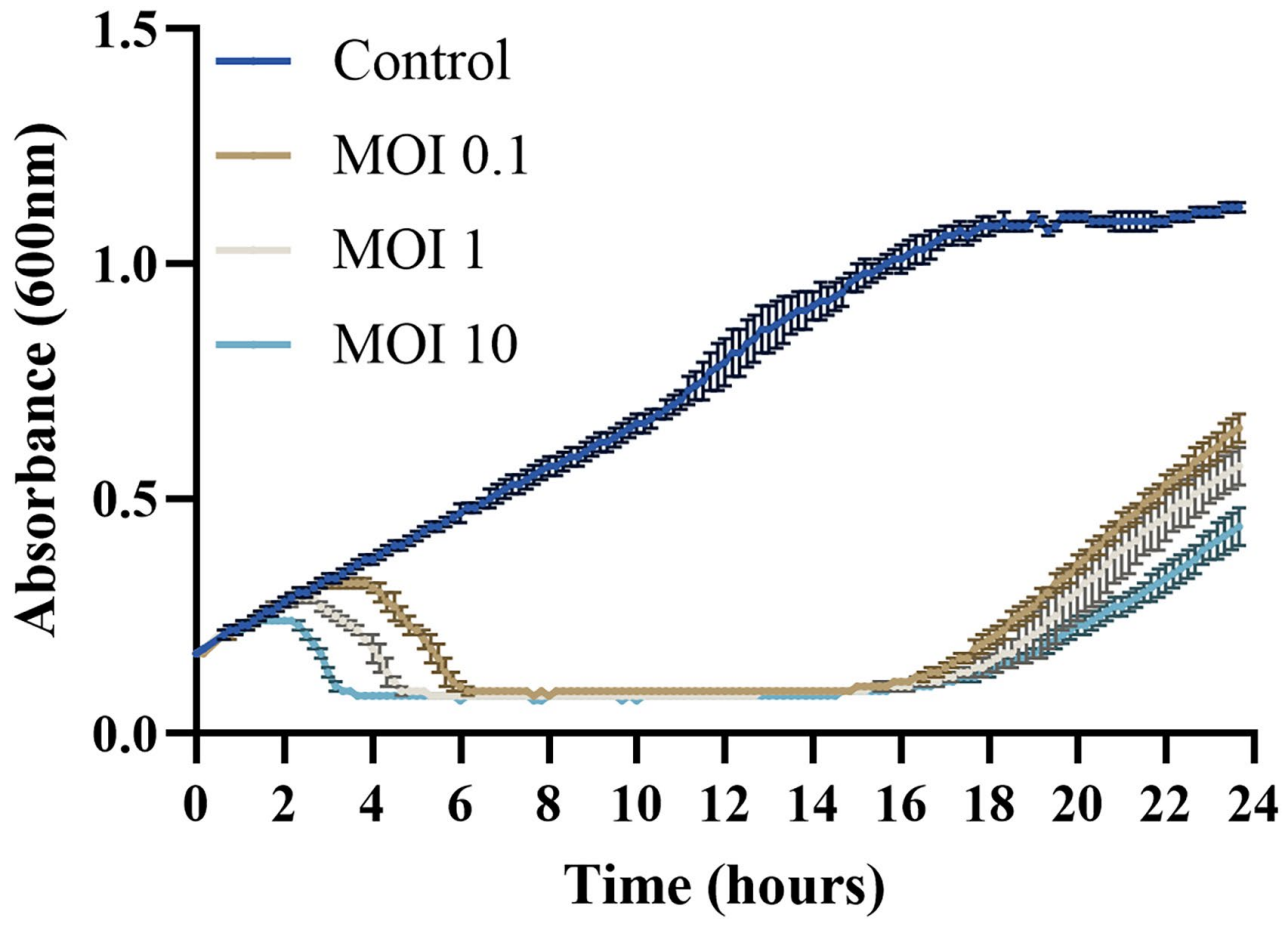
*In vitro* cell lysis assay of Tenacibaculum phage Larrie against host strain LAR25 at three different MOIs (0.1, 1, and 10). Values are mean ± SD (*n* = 3).

The lytic effect of phage Larrie was tested by infecting fresh cultures of the host strain LAR25 at early exponential phage. The results showed that Larrie was able to lyse the bacterial population from MOI 0.1 to 10 after 24 h of incubation. The growth of the bacterium LAR25 was inhibited at 2, 2.5, and 4 h post infection for MOIs 10, 1 and 0.1, respectively, and the reduction was maintained until the end of the experiment (Figure 9).

#### 3.3.3. Genomic features of Tenacibaculum phage Larrie

**Figure 10:**
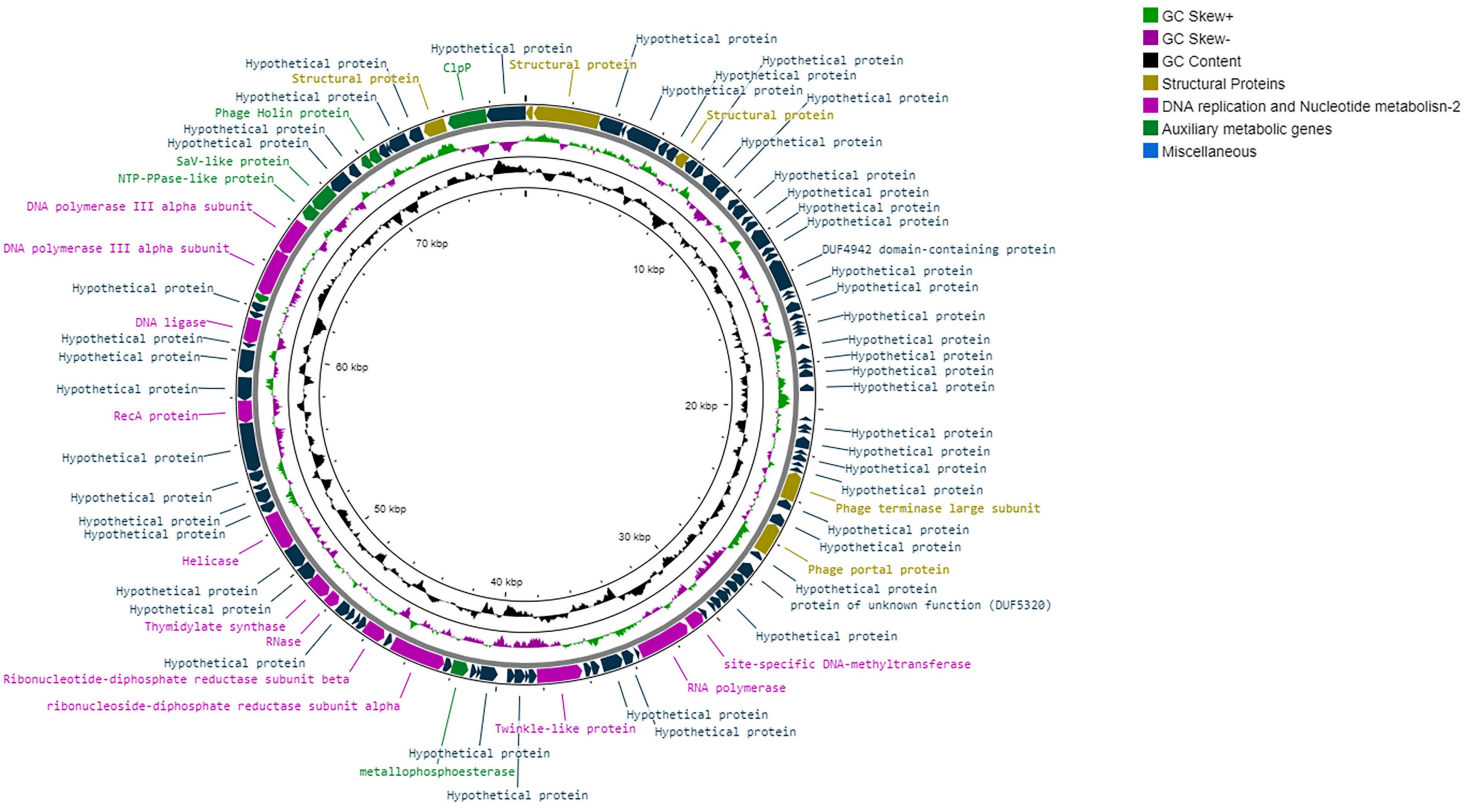
Visual circular representation of Tenacibaculum phage Larrie’s genome. The GC content is represented by the inner black line and the GC skew by the inner purple/green line. The predicted ORFs are presented as arrows. The color of the ORFS refers to annotated biochemical function; phage structural proteins (olive green), DNA replication, repair, and recombination related proteins (purple), miscellaneous proteins (light green), Hypothetical proteins (dark green).

Tenacibaculum phage Larrie has a compact genome of 77,515 bp, with 1.43 genes per kbp with a GC content of 33.7%. Rapid Annotation using Subsystem Technology (RASTk), Glimmer and GeneMark revealed a total of 111 ORFs (Figure 10). Each ORF was manually inspected and validated. No tRNA was present as predicted by ARAGORN tool. ATG was the codon start of 102 ORFs (91.9%), while 7 ORFs had TTG (6.3%) and 2 ORFs had GTG (1.8%). A total of 61 ORFs had significant hits (expected value ≤10^−3^) at the NCBI nr database with an average similarity of 48.84%. Twenty-four ORFs were found to have best hits with Cellulophaga phage phiST (KC821604), while 3 ORFs had best hits with Cellulophaga phage (KC821625) which belongs to the same genus as phiST. A search in the Interpro and NCBI CDD database revealed 15 ORFs and 31 ORFs with significant hits, respectively. No genes related to integration, antibiotic resistance or virulence were detected in Larrie’s genome. As predicted by BACPHLIP, there is a 100% probability that Larrie has a lytic lifestyle, while its genome did not align with any parts of the LAR25 genome. PhageTerm analysis revealed that the genome is terminally redundant and not permuted.

#### 3.3.4. Phage structural proteins

Several proteins associated with phage assembly and structure were identified (ORF 1, ORF 2, ORF 8, ORF 109). A portal protein (ORF 45) and large terminase subunit (ORF 42) required for phage DNA packaging for tailed phages were also present.

#### 3.3.5. DNA replication, repair and recombination

Proteins related to DNA replication, recombination and repair were also identified; RNA polymerase (ORF 56), RNase (ORF 79), DNA or RNA helicase (ORF 62, ORF 83), RecA (ORF 89), DNA ligase (ORF 93) and DNA polymerase subunits (ORF 97, ORF 98). Regulatory elements and proteins involved in nucleotide metabolism were identified at ORF 71, ORF 74, ORF 80, as well as a site-specific DNA-methyltransferase responsible for DNA methylation at ORF 55.

#### 3.3.6. Miscellaneous proteins

A gene coding protein belonging to the protein family of holins was identified at ORF 104 and is involved in bacterial lysis and virus dissemination. Various transmembrane proteins were also revealed at ORF 66, ORF 68, ORF 84, ORF 86, ORF 87, ORF 94, ORF 105. Other miscellaneous proteins, including metallopeptidase (ORF 103), Sav-Like protein (ORF 100), ClpP (ORF 110), gp95 (ORF 96) were identified.

All genomic features of Tenacibaculum phage Larrie annotated together with relevant information based on significant amino acid sequences and protein structural homologies (E-value ≤10^−3^) are provided in Supplementary Table S6.

**Figure 11:**
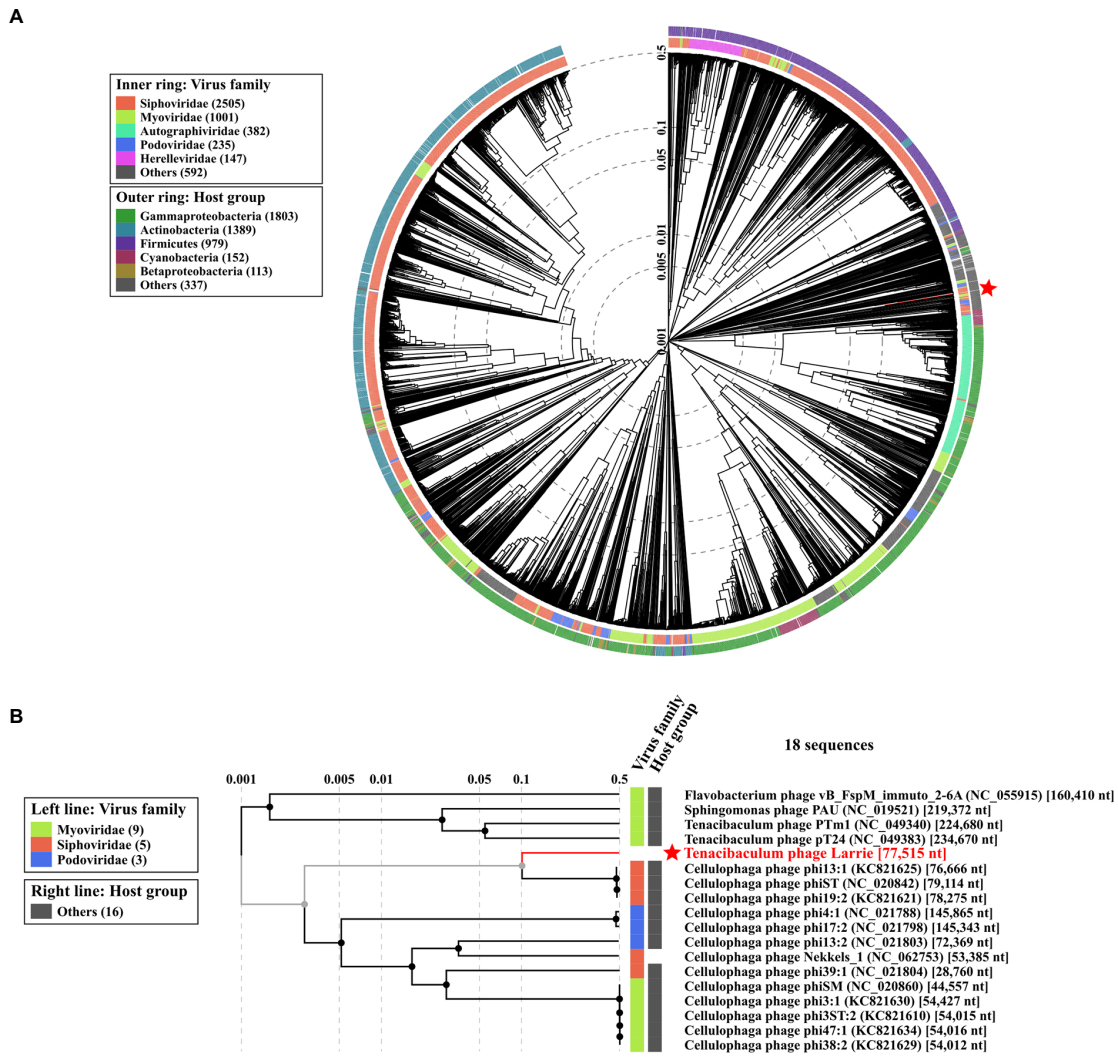
Phylogeny of Tenacibaculum phage Larrie based on proteomic tree constructed by VIPTree. **(A)** Larrie belongs to the Siphoviridae taxonomic family and can infect Flavobacteriaceae group (red star and line). The phage proteome was compared with 4,892 dsDNA phage proteomes (circular tree). The branch length scale was calculated as log values. The inner and outer ring indicate the corresponding taxonomic virus family and host group. **(B)** Below is an excerpt of the tree above showing the phylogeny with only closely related viruses.

**Figure 12:**
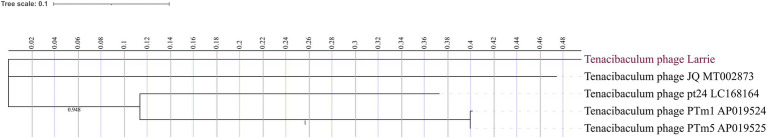
Phylogenetic tree of Tenacibaculum phage Larrie. The large terminase subunits aa sequences of *Tenacibaculum* phages were downloaded from NCBI VIRUS database and aligned using MUSCLE algorithm. A maximum likelihood phylogenetic tree was constructed using MEGA X. The tree was visualized using Interactive Tree of Life (ITOL). The bootstrap value was denoted in each branch.

The phylogenetic analysis of Larrie using the whole proteome in ViPTree showed that it is grouped with other phages infecting hosts of the *Flavobacteriaceae* family with the exception of Shingomonas phage PAU which has as a host *Sphingobacterium paucimobilis*, member of the *Sphingobacteriaceae* (Figure 11). Phylogenetic analysis using the large terminase subunits including all available Tenacibaculum phages is shown in Figure 12. Large terminase subunit gene was used for the phylogeny because it is considered to be a signature, well-conserved gene among the phages and is a strong molecular motor associated with phage packaging (Mitchell, 2002). MAUVE analysis revealed that there was no genomic synteny between phage Larrie and the other phages infecting bacteria of the *Tenacibaculum* genus.

## 4. Discussion

The *Flavobacteriacae* is one of the most ubiquitous families of bacteria in the aquatic environment and its members are key constituents of both freshwater and marine fish hatcheries (Loch and Faisal, 2018; Maapea et al., 2021). The genus *Tenacibaculum* contains several important fish pathogenic species like *T. maritimum, T. dicentrarchi, T. finnmarkense* and *T. discolor*. *Tenacibaculum* *larymnensis* sp. nov. strain LAR25 represents a novel species of the genus since its genomic similarity with the closest related species, *T. discolor* is below 95% which is the accepted threshold for species delineation (Jain et al., 2018). The fatty acid methyl esters profile of *T. larymnensis* sp. nov. is also distinct from the other species of the genus, further supporting its classification as a separate species.

The genomic arsenal of *T. larymnensis* sp. nov. contains several putative virulence factors including secretion systems, metalloproteases, hemolysins and bacterial toxins. One of these virulence genes (LCI24_08180) is predicted to encode a thiol-activated cytolysin which is a pore forming secreted toxin that binds to cholesterol (Rossjohn and Feil, 1997). These toxins are major virulence factors in Gram-positive bacteria but are rarely found in Gram-negative bacteria (Evans et al., 2020). The first discovery of this protein in a Gram-negative bacterium was in *Enterobacter lignolyticus* which is not associated with humans or animals and is not considered a pathogen (Hotze et al., 2013). Thiol-activated cytolysins have also been found in other *Tenacibaculum* spp. (Evans et al., 2020) but their role as virulence factors in this genus has not been investigated so far. The exotoxin ceramidase was also predicted but not along with the sphingomyelinase; however, both act in the same pathway in the pathogenic *T. maritimum* type strain NCIMB 2154 therefore it is not clear if the presence of ceramidase alone would indicate true virulence (Pérez-Pascual et al., 2017). On the other hand, the presence of secretion system proteins like VgrG and PorP/SprF related to T6SS and T9SS, respectively, indicate strong virulent profile. There are many loci attributed to the T9SS, which is associated with the secretion of outer membrane proteins, proteases and gliding motility in the *Bacteroidetes* (McBride and Zhu, 2013; Pérez-Pascual et al., 2017; Saldarriaga-Córdoba et al., 2021). However, the new *Tenacibaculum* species’ pathogenic potential has not been evaluated yet using an *in vivo* challenge assay and it should be underlined that the isolate presented here was not associated to infection or disease.

The versatility of various members of *Flavobacteriacae* to obtain nutrients either as particle-associated or host-associated bacteria is a known trait which is related to the diversity of their enzyme-coding genes. Evidence of this ability is the plethora of carbohydrate-active enzymes (CAZymes) present in its genome which is a typical character of the genus, which thrives in algae blooms (Bunse et al., 2021). *Tenacibaculum larymnensis* was isolated from a hatchery that houses phytoplankton facilities, as well. It possesses 41 CAZymes which is in accordance with what has been reported in the literature for this genus (Gavriilidou et al., 2020).

Another genomic trait that is interesting is the presence of a gene (LCI24_13800) encoding an N-acyl homoserine lactonase, which is a quorum quenching enzyme. A strain of *Tenacibaculum* sp. which is phylogenetically close to *T. discolor* has a demonstrated quorum quenching (QQ) ability against *Edwardsiella tarda* quorum sensing (QS) system (Romero et al., 2014). Since QS apart from a means of communication between bacteria is also an important mechanism of virulence factors expression for many Gram-negative pathogenic bacteria, the disruption of its activity by QQ indicates a putative beneficial role of some of these bacteria as pathogen controllers in the complex environment of the fish hatchery, a hypothesis which has been raised before (Almeida et al., 2021). Quorum quenching activity has been experimentally demonstrated in *Tenacibaculum* sp. strain 20J CECT7426 isolated from marine waters in Spain (Mayer et al., 2015). This strain is genetically closer to *T. discolor*. N-acyl homoserine lactonase is present in various species of *Tenacibaculum* including, *T. maritimum, T. soleae, T. gallaicum, T. aestuarii* and *T. discolor* (data obtained from NCBI accessed on October 12th, 2022). The QS and QQ repertoire vary among the *Tenacibaculum* spp. which provides evidence of the niche adaptation dynamics and different communication demands (Nowlan et al., 2020). Prophage elements are also genomic traits that contribute to bacterial adaptability and evolution. The prophage areas identified by PHASTER represent genomic remnants of phages that had likely infected an ancestral strain of the genus since highly similar areas can also be found in other species like *T. mesophilum* and *T. singaporense* (data not shown). The fact that these phage genes have been retained in the chromosome of the bacterial host is suggestive of a functional role that has yet to be identified. Prophage region 1 contains genes which are characteristic in phage genomes such as phage tail protein and phage holin, while the other two prophage regions contain auxiliary metabolic genes. Interestingly, prophage region 3 contains genes that are related to resistance to oxidative stress including LCI24_13935 encoding TerB family tellurite resistance protein and LCI24_13930 encoding BrxA/BrxB family bacilliredoxin. In *Escherichia coli*, TerB is also located in prophage-like element of the chromosome (Nguyen et al., 2021).

Many anti-phage defense systems were found in the LAR25 genome. Apart from several R-M systems commonly found in bacteria, CBASS II system, Mokosh type II system, Gabija and Septu systems were also present. CBASS II system is an Abi (abortive infection) system that leads to host cell death to avoid phage propagation (Duncan-Lowey and Kranzusch, 2022), while Mokosh type II involves the recognition of phage RNA possibly to disrupt phage replication (Doron et al., 2018). Gabija and Septu offer phage resistance through nucleotide regulation (Doron et al., 2018). The co-occurrence of these anti-phage systems, whether they synergistically provide anti-phage defense or not, predominantly depends on the pressure of selection (Wu et al., 2022) and is something worthy of further investigation.

The *Tenacibaculum* genus is a significant component of marine hatchery environment especially in the Mediterranean area (unpublished data from our group). Although the genus contains several pathogenic species, many members can be avirulent environmental bacteria. It should be underlined that the novel species presented here was not a clinical but an environmental isolate. Bacterial communities established in a commercially hatchery environment can be exposed to various antibiotics since bacterial disease treatments although not common at the early developmental stages of the fish are applied when necessary. The analysis resulted in the prediction of few antibiotic resistance genes (ARGs) for 4 antibiotic classes. Tetracycline is the main antibiotic used in Greek aquaculture (Rigos and Kogiannou, 2023), and tetracycline resistance genes are common among isolates of the Greek fishfarm environment (Nikolakopoulou et al., 2008), thus the presence of *tetM* and the phenotype of intermediate susceptibility of the strain are correlated and expected. Antibiotics used to target these pathogens can be enrofloxacin, florfenicol, trimethoprim and sulfadiazine (Nowlan et al., 2020; Rigos et al., 2020; Saldarriaga-Córdoba et al., 2021) and no ARGs were detected for them. Resistance to oxolinic acid has been reported for *T. maritimum, T. piscium* and *T. finnmarkense* (Avendaño-Herrera et al., 2008; Olsen et al., 2020). *T. maritimum* was resistant to oxolinic acid (2 μg) and susceptible to flumequine (30 μg), that agrees with LAR25 results. Antibiotic treatment is the only way to combat *Tenacibaculum* sp. in Greek aquaculture, as there is no commercial vaccine, and juvenile fish suffer occasionally from tenacibaculosis in the hatchery environment (Rigos et al., 2020), therefore alternative approaches such as phage therapy are needed.

Tenacibaculum bacteriophage Larrie is a novel bacteriophage belonging possibly to a novel genus since no genomic similarity at the nucleotide level with any other bacteriophage included at the NCBI database (accessed on October 12, 2022) was found. Larrie is a siphovirus and showed strong lytic activity against its host, while the inability to infect any of the other *Tenacibaculum* species tested is suggestive of a narrow host range, which however can be a bias caused by the small number of strains tested. Until today, only five phages infecting *Tenacibaculum* spp. have been isolated and only two of them have been characterized. The jumbo phages PTm1 and PTm2, both having as host *T. maritimum* were isolated from a netpen fish farm in Japan (Kawato et al., 2020). They are similar Myoviruses with large genomes (224–226 kb) and peculiar hair-like appendages protruding from the head. Interestingly, phylogenetic analysis using the whole proteome of all available phages in VIPtree showed that Larrie is clustered together with Cellulophage phages (Holmfeldt et al., 2013) which form a separate clade to the clade formed by the five phages infecting *Tenacibaculum* spp. The similarity between Cellulophaga Phi13:1 and phage Larrie is likely due to the fact that both Phi13:1 and Larrie are siphoviruses and are structurally more similar. Genes that encode structural proteins tend to be more conserved among phages in the context of the genetic mosaicism that characterizes their genomes. *Cellulophaga* spp. have an important ecological role because they are also efficient degraders of polysaccharides and are particularly significant for phytoplankton and algal cell wall lysis.

Bacteriophages shape the microbial communities in the ocean, but this is also the case in more confined environments like those in aquaculture (Hoikkala et al., 2019). Virulent bacteriophages contribute to the microbial diversity of the aquaculture environment by the selective pressure they apply to dominant species as the Kill-the-Winner model hypothesizes (Chevallereau et al., 2022). This can be particularly relevant in situations where nutrients availability is high promoting bacterial competition for these resources favoring the dominance of fast growing and efficient/fit bacteria. In this scenario, predation of the dominant bacteria by lytic bacteriophages limits significantly their dominance offering the possibility to less fit bacterial species to participate in the microbial community. Well-characterized bacteria-phage systems can have a pivotal role in studying the microbial population dynamics especially when using aquaculture settings as a study habitat which is of course less complex and steadier than the open sea but at the same time not as simplified as the limited laboratory conditions (Hoikkala et al., 2019). To this end, the novel fully characterized *Tenacibaculum* phage-host system can serve as an efficient model to study microbial interactions in the aquatic environment which contribute to the nutrient cycling.

Phage therapy has re-emerged the past years as a promising alternative to antibiotics and as a means to overcome the issue of antimicrobial resistance (Gordillo Altamirano and Barr, 2019). Aquaculture is considered a hotspot for the development of antimicrobial resistance (Watts et al., 2017) and the reduction of antibiotic usage is required urgently. Moreover, at the hatchery environment where a delicate microbial equilibrium is extremely important for the healthy development of the juveniles, the use of antibiotics is rather problematic since it affects microbial populations indiscriminately. In such cases, a targeted treatment like phage therapy would be more suitable (Katharios et al., 2017). Although this novel *Tenacibaculum* species cannot be considered as a pathogen, the successful isolation of a phage with demonstrated lytic capacity against it, corroborates previous reports (Kawato et al., 2020) that phage therapy could also be applied to members of this genus.

## Data availability statement

The datasets presented in this study can be found in online repositories. The names of the repository/repositories and accession number(s) can be found in the article/Supplementary material.

## Author contributions

PK designed the study and performed electron microscopy. MT, CK, and SD performed microbiological analyses. AT, PK, and SD performed bioinformatic analysis. MT, AT, CK, SD, and PK interpreted the results, drafted the sections of the manuscript, prepared the figures and tables, and prepared the final version of the manuscript. AT and PK critically revised the manuscript. All authors read, reviewed, and approved the manuscript.

## Funding

This work was funded by the project MIS 5010932: OP Fisheries and Maritime 2014–2020 Innovative measures, Innovation 2019.

## Conflict of interest

Author GA was employed by PHILOSOFISH S.A.

The remaining authors declare that the research was conducted in the absence of any commercial or financial relationships that could be construed as a potential conflict of interest.

## Publisher’s note

All claims expressed in this article are solely those of the authors and do not necessarily represent those of their affiliated organizations, or those of the publisher, the editors and the reviewers. Any product that may be evaluated in this article, or claim that may be made by its manufacturer, is not guaranteed or endorsed by the publisher.

## Footnotes

^1^ http://www.geneious.com

^2^ https://proksee.ca/
